# Influence of spatial-intensity contrast in ultraintense laser–plasma interactions

**DOI:** 10.1038/s41598-022-05655-4

**Published:** 2022-02-03

**Authors:** R. Wilson, M. King, N. M. H. Butler, D. C. Carroll, T. P. Frazer, M. J. Duff, A. Higginson, R. J. Dance, J. Jarrett, Z. E. Davidson, C. D. Armstrong, H. Liu, S. J. Hawkes, R. J. Clarke, D. Neely, R. J. Gray, P. McKenna

**Affiliations:** 1 SUPA Department of Physics, University of Strathclyde, Glasgow, G4 0NG UK; 2 The Cockcroft Institute, Sci-Tech Daresbury, Warrington, WA4 4AD UK; 3 Central Laser Facility, STFC Rutherford Appleton Laboratory, Oxfordshire, OX11 0QX UK; 4 Beijing National Laboratory for Condensed Matter Physics, Institute of Physics, Chinese Academy of Sciences, Beijing, 100190 China

**Keywords:** Laser-produced plasmas, Plasma-based accelerators

## Abstract

Increasing the intensity to which high power laser pulses are focused has opened up new research possibilities, including promising new approaches to particle acceleration and phenomena such as high field quantum electrodynamics. Whilst the intensity achievable with a laser pulse of a given power can be increased via tighter focusing, the focal spot profile also plays an important role in the interaction physics. Here we show that the spatial-intensity distribution, and specifically the ratio of the intensity in the peak of the laser focal spot to the halo surrounding it, is important in the interaction of ultraintense laser pulses with solid targets. By comparing proton acceleration measurements from foil targets irradiated with by a near-diffraction-limited wavelength scale focal spot and larger F-number focusing, we find that this spatial-intensity contrast parameter strongly influences laser energy coupling to fast electrons. We find that for multi-petawatt pulses, spatial-intensity contrast is potentially as important as temporal-intensity contrast.

## Introduction

Since the first demonstration of lasing, there has been a continual drive to increase the achievable peak intensity of laser light, and in the case of solid state lasers this has resulted in an average increase of two to three orders of magnitude per decade. Intensities in the range $10^{18}$–$10^{20}\,\hbox {W cm}^{-2}$ are now routinely achieved at high power laser facilities worldwide^1^ and this has opened up new scientific topics such as relativistic optics and photonics^2^, laser-driven particle^3^ and radiation sources^4^, and high field science^5^. Laser intensity is a key parameter in laser–solid interactions as it strongly influences the motion of electrons in the laser field and the temperature to which plasma electrons are heated, and by extension, defines many of the properties of beams of laser-generated ions^6, 7^, X-rays^8, 9^ and high harmonics^10, 11^. Laser intensity can be enhanced by increasing the pulse energy, decreasing the pulse duration or focal spot size, or by a combination of these approaches. Due to the damage threshold of optical materials and coatings, increasing the energy requires that the size of the beam, and thus the size of expensive optical components, also increases. In recent years, efforts to boost the intensity have therefore focused on generating shorter pulses or achieving tighter focusing. The latter, due to the costs involved in producing large diameter short focal length optics and potential damage from target debris, has led to innovative approaches, such as the use of disposable focusing plasma mirrors (FPM)^12–14^. These efforts and the employment of extremely tight focusing optics drive the need to understand how the resulting decrease in focal spot size influences the laser–plasma interaction physics and properties of the secondary sources of energetic particles and radiation.

Previous studies on the role of laser focal spot diameter show that it, in part, defines properties of the fast electron population generated at the focus and accelerated into the target^15–17^. As a result, it also affects the properties of the beams of particles and radiation generated by those electrons. In the case of beams of ions accelerated by the target normal sheath acceleration (TNSA) mechanism^7^, it has been demonstrated to change the energy spectrum, spatial distribution and laser-to-ion energy conversion efficiency^18–23^.

As multi-petawatt laser facilities become operational and the peak power of laser pulses continues to increase, not only is the influence of the main laser focal spot size important, but so too is the intensity of the light in the halo region at larger radii (the *wings* surrounding the main spot). Relativistic electron motion occurs when the normalised laser amplitude $a_{0} \ge 1$, where $a_{0}=eE_{0}/(\omega _{L} m_e c)$, $m_e$ is the electron rest mass, *c* the speed of light, *e* is the electron charge, and $E_{0}$ and $\omega _{L}$ are the laser electric field amplitude and angular frequency, respectively. For an idealised focal spot (i.e. Airy disk intensity distribution), when the main peak is of ultrahigh intensity ($a_{0} > 100$), the intensity in the wings will be above the threshold for relativistic effects and could therefore play a significant role in the interaction. For a laser wavelength $\lambda _{L}= 1 \,\upmu \hbox {m}$, $a_{0} = 1$ occurs at $\sim 10^{18}\,\hbox {W cm}^{-2}$. Additionally, the small *F*-number optics used to achieve tight focusing are more susceptible to laser wavefront aberrations and alignment sensitivity, which typically results in reduced focal spot quality and increased energy in the wings. The importance of considering the energy in the wings, in the accurate calculation of the main focal spot intensity, has been considered, for example in Hartmann et al.^24^, but the influence of this on laser-matter interaction physics has, to our knowledge, not been explored to date.

**Figure 1:**
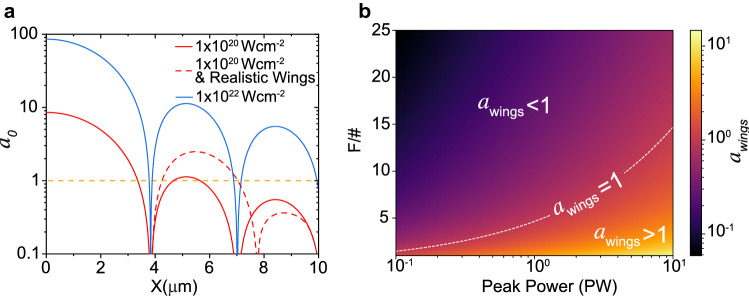
Conditions for which the intensity in the wings is relativistic. (**a**) Normalised vector potential ($a_{0}$) as a function of radius, for idealised (Airy disk) focal spots at two given peak intensities, each of the same diameter (FWHM). The dashed red data illustrates a case for which the intensity distribution is non-ideal, resulting in a higher intensity in the wings (a degradation of the spatial-intensity contrast). The dashed orange line marks the relativistic threshold intensity for $1\, \upmu \hbox {m}$ light. (**b**) Normalised vector potential of the laser light in the wings, $a_{wings}$, as a function of laser pulse power and focusing geometry. The $a_{wings}=1$ curve marks the threshold for which the intensity in the wings is relativistic.

Analogous to the concept of temporal-intensity contrast, which is the ratio of the peak laser intensity to the intensity of the light at a given time before or after the peak (which strongly influences expansion of the plasma before the main pulse arrives), one can consider spatial-intensity contrast to be the ratio of the peak intensity to the intensity of the light at a secondary spatial location or defined area in the wings. Figure 1a illustrates the concept, for idealised focal spot intensity profiles (in terms of $a_{0}$) at two peak intensities and a case where the spot spatial-intensity profile is sub-optimal, resulting in more laser energy in the spot wings. It is clear that for the case of ultrahigh peak intensities of the order of $\sim 10^{22}\, \hbox {W cm}^{-2}$, the intensities in several Airy disks are considerably above the relativistic threshold and even at $\sim 10^{20}\,\hbox {W cm}^{-2}$ for the case of an non-ideal distribution (no particular significance to the shape chosen), the intensity in the first Airy disk can be significantly above the relativistic threshold. The result of this is significant generation of relativistic electrons on spatial scales well outside the central intense focal spot. To illustrate this further, Fig. 1b presents the corresponding value of the light amplitude vector in a defined area in the wings ($a_{wings}$) as a function of focusing *F*/# and laser power parameter space. In this example case, the area of the wings is a fixed factor ($\times 20$) larger than the area defined by the peak full-width half-maximum (FWHM) and the ratio of the energy in the wings to the energy in the main focal spot is fixed. It is clear that at multi-petawatt power levels, the light intensity in the wings will be relativistic over a wider range of focusing geometries.

Here we report on an experimental and numerical investigation examining the influence of focal spot spatial-intensity contrast on laser–solid interactions. A tight focusing geometry is used and the properties of the beam of protons accelerated by the TNSA mechanism are measured as a function of the laser intensity in the wings. We find a $\times 2.7$ enhancement in the laser-to-proton energy conversion efficiency when compared to the case of a larger focal spot with a much lower intensity in the wings, for an equivalent peak laser intensity. Through the use of analytical modelling and 2D particle-in-cell (PIC) simulations, we establish that the relativistically intense light in the wings generates an additional population of fast electrons at the target front surface, which contribute to the generation of the sheath field at the target rear and the subsequent proton acceleration. Although proton acceleration is explored as a diagnostic of the change to the interaction physics, the additional fast electrons injected into the foil affects a wide range of physics including the generation of high harmonics, X-rays and THz radiation. We introduce a simple model to define the parameter space for which the light surrounding the central focal spot contributes significantly to the interaction physics.

## Results

### Role of focal spot spatial-intensity contrast on laser-driven proton acceleration

**Figure 2:**
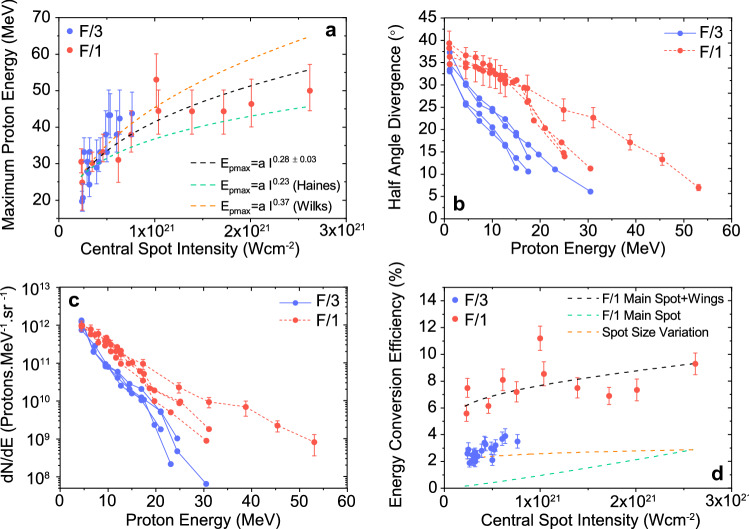
Measured proton beam properties for two different focusing geometries. (**a**) Maximum proton energy as a function of the central spot peak intensity ($I_L$). The dashed black curve is a power law fit to the measurements. The orange and green dashed curves represent the $E_{pmax}$ scaling as modelled using the Mora^28^ formulae with the hot electron temperature determined from the Wilks^29^ and Haines^30^ models, respectively. (**b**) Representative measurements of proton beam divergence as a function of energy. (**c**) Same for proton beam energy spectra. In (**b**) and (**c**), the peak intensity in each focusing case is approximately equal ((3 ± 1) $\times 10^{20}$ $\hbox {W cm}^{-2}$), with the exception of the highest $E_{pmax}$ F/1 data, for which the intensity was 1 $\times 10^{21}$ $\hbox {W cm}^{-2}$. The representative error bars are applicable to all of the data. (**d**) Laser-to-proton energy conversion efficiency as a function of $I_L$. Results of a simple model of the efficiency scaling for three cases: (i) F/1 spot, including wings (dashed black); (ii) F/1 central spot only (dashed green); and (iii) varying central spot size (dashed orange). In all cases, 6 $\upmu$m-thick Al foil targets were irradiated. Each data point in (**a**) and (**d**) represents a single laser shot.

To determine the influence of spatial-intensity contrast, Fig. 2 compares measured properties of the beam of accelerated protons from the irradiation of $6\,\upmu \hbox {m}$-thick aluminium target foils using F/1 and F/3 pulse focusing geometries. The laser intensity, $I_L$, range in the F/1 data ($2.3\times 10^{20}$–$2.6\times 10^{21}\,\hbox {W cm}^{-2}$) is predominately the result of changes in the ratio of the energy in the main focal spot FWHM and the energy at larger radii (in the wings), as discussed in the “Methods” section.

Figure 2a shows the measured maximum proton energy, $E_{pmax}$, as a function of $I_L$. In contrast to other beam properties, as discussed below, there is no measurable difference between the $E_{pmax}$ values for the two focusing geometries for a given equivalent intensity, indicating that the energy in the wings, which is substantial at the lower intensity end of the F/1 dataset, does not play a significant role in defining the maximum proton energy. Dover et al.^25^ report less effective proton acceleration, in a shorter pulse regime ($\sim 40 \,\hbox {fs}$), when the laser focal spot size approaches the diffraction limit (i.e near-wavelength size), in which electron transverse motion can be larger then the focal spot size, but this is not observed for the longer pulse case considered here.

Overall, $E_{pmax}$ is tending to saturate in the upper region of the intensity range ($>10^{21}\,\hbox {W cm}^{-2}$). A power law fit of the form $E_{pmax}=a \cdot I_L^{b}$ over the full intensity range (including the F/3 and F/1 measurements) results in the exponent $b=(0.28\pm 0.03)$. This scaling with laser intensity is less favourable than previous measurements at lower intensities with the same laser and pulses focused to a larger focal spot, for which $b\approx 0.5$^26^. A similar behaviour is reported in Nakatsutsumi et al.^27^ for pulse durations of a similar length, and attributed to magnetic fields self-generated on the target rear surface inhibiting sheath acceleration. However, even in the absence of such effects, we find that our measurements can be explained by changes to the fast electron temperature—the pulse energy is very similar across the intensity range and so as the electron temperature increases, the number of electrons generated, and thus electron density, decreases (in contrast to the $b\approx 0.5$ scaling which was obtained by varying the laser pulse energy to change the intensity^26^). Using the 1D plasma expansion model introduced in Mora^28^, with the fast electrons spectrum assumed to be described by the electron temperature scalings presented in Wilks et al.^29^ and Haines et al.^30^, power scaling exponents of $b=0.37$ and $b=0.23$ are obtained, respectively. Our measurements are between the predictions of these two temperature scaling models, as shown in Fig. 2a.

Clear differences are observed in the measured proton beam spatial divergence as a function of proton energy for the two focusing cases, as shown in the representative measurements presented in Fig. 2b. Consistent with previous results, the divergence decreases with increasing proton energy as a result of the radial variation in the sheath expansion dynamics (the area over which protons are sourced decreases with increasing proton energy^31, 32^). However, the proton beam obtained with F/1 focusing is clearly more divergent over the full energy range than that produced by F/3 focusing. The average reduction in beam divergence between the two is a factor of $\times 1.2$ at 4.5 MeV, and $\times 2$ at 17.5 MeV.

Representative proton energy spectra measured using RCF spectroscopy, are shown in Fig. 2c. Over most of the energy range the proton numbers (per steradian) produced with F/1 are significantly higher than for F/3 and the overall proton beam temperature is also higher. Temperature fits to the data results in average temperatures of 8.6 MeV and 6.5 MeV for the F/1 and F/3 cases, respectively. The corresponding differences in the laser-to-proton energy conversion efficiency as a function of $I_L$ are shown in Fig. 2d. For the intensity range for which equivalent $I_L$ is achieved with both focusing geometries, F/1 focusing results in an average of a factor of $\times 2.7$ higher conversion efficiency compared to F/3. There is significant energy in the wings (up to 90% of $E_L$ for the lowest $I_L$) for the F/1 case. To test for the influence of both the focal spot size and energy in the wings, the proton beam properties produced by both components of the laser focus distribution are modelled using the Mora^28^ plasma expansion formulae (with the acceleration time truncated at $\times$1.3 of the laser pulse duration, as introduced by Fuchs et al.^33^). Using measurements of the encircled energy within the focal spot FWHM and the wing diameter for the F/1 FPM (see “Methods”), the proton spectrum expected from both components are calculated separately, and the total proton energy summed to calculate the overall proton number on-axis. The 1D model does not take account of the 2D distribution of the proton beam and thus cannot be used to predict the total energy coupled to protons or divergence. The model results shown in Fig. 2d are normalised to the highest intensity experimental F/1 value and the same normalisation factor is applied to all three model results. The scaling of the measured values with $I_L$ is reproduced when including the protons accelerated by both the central focal spot and the wings (black curve). The values are significantly lower when the energy in the wings is not included (green curve) or when just the central focal spot size is varied (orange curve). The conversion efficiency is observed to strongly depend on the total focal spot spatial intensity distribution under these focusing conditions.

The observed differences in the proton beam divergence for the two focusing cases (Fig. 2b) suggest differences in the sheath field distribution^34^. As discussed in Carroll et al.^35^, a near-linear dependence of divergence on proton energy as measured for F/3 results from an inverse parabolic sheath profile, whereas the more slowly decreasing divergence with energy as measured for F/1 is more consistent with a Gaussian shaped sheath distribution, i.e. with protons accelerated at larger radii. The fact that the numbers of protons accelerated and thus the conversion efficiency are significantly higher for F/1 is consistent with a larger sheath area.

### Numerical modelling of the role of spatial-intensity contrast

**Figure 3:**
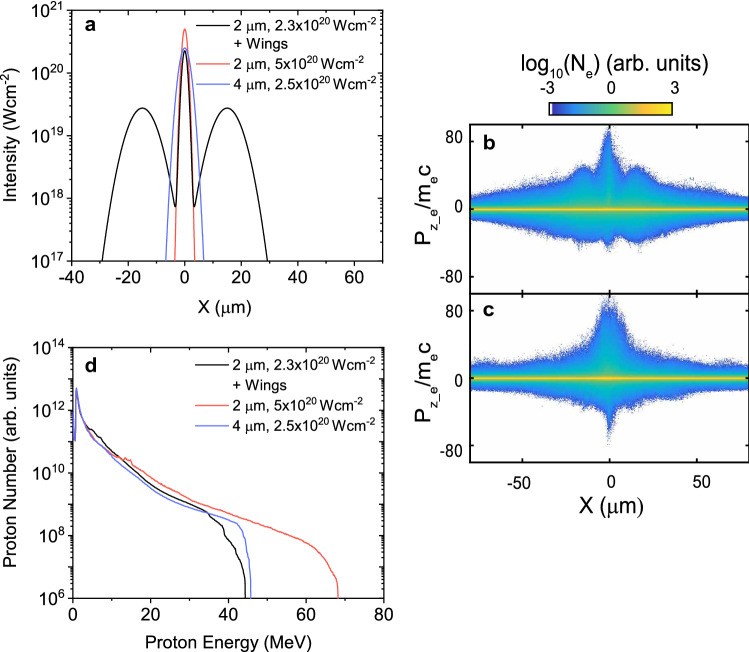
Simulation results illustrating the effects of the focal spot wings. (**a**) Example input focal spot spatial-intensity profiles for the 2D PIC simulations, for given focal spot diameter, intensity and profile conditions. (**b**) Longitudinal momentum of all electrons obtained 100 fs after the peak of the laser interaction, shown as a function of the transverse position (the laser is incident at $\hbox {X}=0$), for a $2\,\upmu \hbox {m}$ diameter focal spot including wings. (**c**) Same for a $4\,\upmu \hbox {m}$ diameter spot without wings. (**d**) Example proton energy spectra obtained for given focal spot diameter, intensity and profile conditions.

In order to investigate changes to the sheath dynamics and proton acceleration arising from varying the focal spot size and spatial-intensity distribution, 2D PIC simulations were conducted using the fully relativistic PIC code EPOCH^36^ (see “Methods” section). The focal spot spatial profile was varied in two ways, with the total laser pulse energy fixed in all cases. Firstly, the diameter (FWHM), *d*, of the Gaussian profile central spot was varied from 2 to $5 \,\upmu \hbox {m}$ with the corresponding intensity ranging from $5\times 10^{20}\, \hbox {W cm}^{-2}$ to $2\times 10^{20}\,\hbox {W cm}^{-2}$, ensuring energy conservation in 2D (due to there being only one transverse dimension). In the second scan, to model the effect of the wings, the Gaussian profile central spot with $d=2 \,\upmu \hbox {m}$ was combined with satellite peaks with $d=10 \,\upmu \hbox {m}$, one either side of the central axis and with their centre offset from the axis by $15 \,\upmu \hbox {m}$. The ratio of the energy in the central spot to the wings was varied between 0.3 and 1, resulting in a comparable variation in peak intensity and again conserving energy. Figure 3a shows examples of the focal spot intensity profiles used in the simulations.

It is found that as the transverse spatial profile of the focal spot is increased, due to inclusion of the wings, a greater number of energetic electrons are generated further from the central laser axis. This is observed in Fig. 3b,c, which shows the longitudinal momentum density maps of all electrons, obtained 100 fs after the peak of the laser interaction, as a function of the transverse position (the laser central axis is along X = 0). It is shown for a central spot of $2\,\upmu \hbox {m}$ diameter including wings (Fig. 3b) and $4\,\upmu \hbox {m}$ diameter with no wings (Fig. 3c), with the same total laser energy. This leads to differences in the proton acceleration at the target rear side (explored in detail below), with example proton energy spectra presented in Fig. 3d. The total proton energy is higher for the case including the wings.

**Figure 4:**
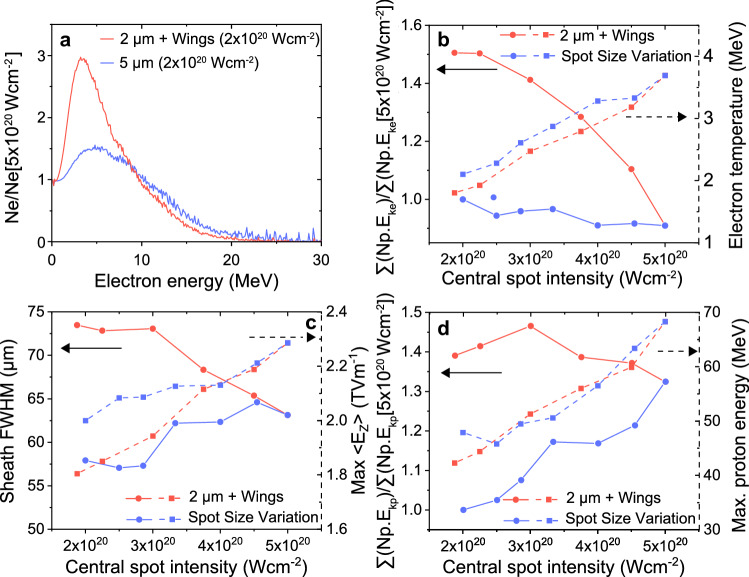
Simulation results illustrating the influence of spatial-intensity contrast. (**a**) Electron spectra for a central spot intensity of $2\times 10^{20}\, \hbox {W cm}^{-2}$ normalised to the corresponding spectra for $5\times 10^{20}\,\hbox {W cm}^{-2}$ at $d=2\,\upmu \hbox {m}$, for an example case in each of the central spot diameter (*d*) and wing energy ($d=2\,\upmu \hbox {m}$) scans. (**b**) Total energy in the fast electron population (solid), normalised to the corresponding values for the $d=5\,\upmu \hbox {m}$ focal spot case, and electron temperature (dashed). (**c**) Transverse size FWHM (solid) and maximum longitudinal electric field strength (dashed) of the spatially averaged rear surface sheath field. (**d**) Total energy of the accelerated protons (solid), normalised to the $5\,\upmu \hbox {m}$ focal spot case, and maximum proton energy (dashed). In (**b–d**) plots are as a function of central spot intensity changed by varying *d* (blue) and wing energy content for $d=2\,\upmu \hbox {m}$ (red). The total laser pulse energy is fixed in all cases.

Figure 4 summarises the effect in greater detail. When *d* is increased (reducing intensity) without the presence of wings, the number of high energy electrons is observed to decrease, whilst the lower energy electron population increases due to the increase in the area of the target irradiated. This can be seen in the example case of the ratio of the electron spectra of a $d=2\,\upmu \hbox {m}$ spot ($5\times 10^{20}\,\hbox {W cm}^{-2}$) to a $d=5\,\upmu \hbox {m}$ spot ($2\times 10^{20}\,\hbox {W cm}^{-2}$) shown in Fig. 4a (blue curve). When the central spot size is fixed at $d=2\,\upmu \hbox {m}$ and the energy is transferred to the wings (red curve in Fig. 4a) a similar effect is observed for the same peak intensity, but significantly more lower energy electrons are produced. This is due to the larger interaction area arising from the addition of the wings, which significantly increases the coupling of laser energy into electrons, as observed when comparing the solid red and blue curves in Fig. 4b. By contrast, the fast electron temperature decreases in a similar way for both scans as the central spot intensity is reduced, demonstrating the strong correlation with the peak intensity of the central spot. The total energy in the fast electron population is determined by integrating the electron energy spectrum above 1 MeV, across the whole simulation box, 100 fs after the interaction of the pulse peak temporal intensity. The electron temperature is determined by fitting to the hot electron tail of the same spectrum (i.e. above 4 MeV).

Both the number and temperature of the fast electrons play an important role in defining the sheath field at the rear of the target. The increased coupling of energy to the electrons due to the presence of the wings results in an increase in the size of the sheath at the rear of the target, as shown in Fig. 4c, whereas the maximum strength of the field follows a trend similar to the changes in the fast electron temperature (i.e. is defined by the central spot intensity).

The increase in the transverse extent of the sheath field means that more protons are accelerated, and this is shown in Fig. 4d, in the comparison of total energy of the accelerated protons for both scans. The total proton energy is sampled 500 fs after the peak of the pulse interacts with the target for both methods of varying intensity. To account for the 2D nature of the simulations, the measure of the energy content was extrapolated to 3D by assuming cylindrical symmetry and scaling each particle weight by $2\pi X$, where *X* is the particle position in the transverse direction. The efficiency values are normalised to the total energy for $d=5\,\upmu \hbox {m}$ (the case which approximates the *F*/3 experimental condition at $2\times 10^{20}\,\hbox {W cm}^{-2}$). The maximum proton energy, by contrast, is very similar for both scans and is primarily defined by the fast electron temperature.

Overall, and consistent with the experiment trends, we find that the total energy of the accelerated protons is strongly determined by the effective area and spatial energy distribution of the laser spot, although these parameters have much less influence on the maximum proton energies achieved.

### Analytical model of the wing conditions influencing the interaction

We next introduce a simple model to aid in determining the conditions for which the light level in the wings generates relativistic electrons and contribute significantly to the laser–plasma interaction physics. This will be the case when the normalised vector potential (defined above) for the light in the wings is $a_{wings} >1$. Using the definition of intensity, $I=(\epsilon _{0} c/2)|E|^2$, where $\epsilon _{0}$ is the permittivity of free space and grouping the constants ($2\pi ^2 m_e^2 c^5 \epsilon _{0}/e^2=1.37\times 10^{10}\,\hbox {W}$), the threshold intensity at $a_{wings}=1$ can be defined as:

1 $$\begin{aligned} I_{(a_{wings}=1)}\approx \frac{1.37\times 10^{10} [\texttt {W}]}{\lambda _L^2}.\end{aligned}$$

To estimate the wing intensity, $I_{w}$, the energy content of the wings, $E_{w}$, can be related to the total pulse energy, $E_{L}$, and the central spot encircled energy fraction, $\eta$, through $E_{w}=(1-\eta )E_{L}$ and the spatial area of the wings, $A_{w}(\zeta )$, where $\zeta$ represents the spatial distribution of the wings, dependent on a beam line specific term (or number of terms), including focusing geometry, alignment and wavefront aberrations. This can be expressed as:

2 $$\begin{aligned} I_{w}=\frac{(1-\eta )E_{L}}{\tau _LA_{w}(\zeta )}, \end{aligned}$$

where $\tau _L$ is the pulse length. For simplicity, it is assumed the duration of the wings is the same as the main pulse, though this may not be the case if there are wavefront aberrations. Finally, equating both and rearranging for $\eta$, gives the central spot encircled energy value, for a given arbitrary wing area function, where $a_{wings}=1$;

3 $$\begin{aligned} \eta =1-\frac{1.37\times 10^{10}[\texttt {W}]\tau _L A_{w}(\zeta )}{E_{L}\lambda _L^2}. \end{aligned}$$

**Figure 5:**
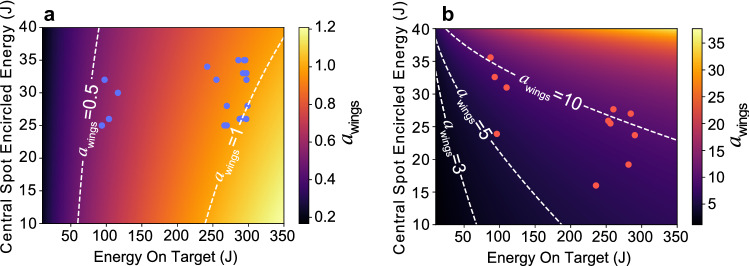
Model results for the normalised vector potential of the wings for both focusing geometries. Calculated values for $a_{wings}$ (colour axes) as a function of peak encircled energy in the main spot (FWHM) and the total laser pulse energy on-target, for (**a**) F/3 focusing and (**b**) F/1 focusing. Both are based on the focal spot distributions measured in the experiment. The calculated values for the specific focal spot distributions for the measurements in Fig. 2 are included as data points.

Figure 5a and b show the calculated values of $a_{wings}$ as a function of central spot encircled energy and energy on target, demonstrating the *F*/3 and *F*/1 experimental conditions for which $a_{wings} \geqslant$1, respectively. In Fig. 5a, $A_{w}(\zeta )$ is selected such that the wings are $\times 40$ larger in diameter than the central focal spot size, based on measurements of the spatial-intensity distribution of the *F*/3 focal spot in the experiment. In Fig. 5b, this function is formed based on the *F*/1 FPM wing characterisation (described in the “Methods” section), and used in the analytical modelling of the laser-to-proton energy conversion efficiency to estimate the wing conditions in Fig. 2d. For the *F*/1 focusing case, $5\leqslant a_{wings} \leqslant 11$, which would result in a significant increase in the coupling to fast electrons and subsequently to protons over the *F*/3 focusing case, for which $a_{wings} \leqslant 1$.

## Summary

The influence of focal spot spatial-intensity contrast in ultraintense laser–solid interactions is explored. Through measurement of the properties of the beam of accelerated protons, it is shown that the intensity in the focal spot wings of a tightly focused laser pulse, arising from effects such as focusing aberrations, strongly influences the overall laser energy coupling to fast electrons. Analytical modelling and 2D PIC simulations show that when the intensity in the wings is above the relativistic threshold, the spatial region at the front of the target over which fast electrons are generated is larger, which in turn increases the area at the target rear side over which a sheath electric field is formed and thus the overall proton source area. Although this has little influence on the maximum proton energy (which is defined by the peak intensity), it strongly defines the overall energy coupling to electrons and protons, as well as the divergence properties of the proton beam.

As the efficiency of laser energy coupling to electrons and the properties of the resultant beam of fast electrons injected into the target are key factors in most laser–solid interaction topics, this study highlights the importance of spatial-intensity contrast as the field moves towards higher peak intensities achievable at multi-petawatt laser facilities. It is imperative that this parameter, which has received scant attention to date, is accurately characterised and considered in experiment design, modelling and the interpretation of measurements using these new facilities. It also merits consideration when analysing hitherto unexplained experimental results at the intensities achievable at present. Shot-to-shot variability in the pulse wavefront, which alters the ratio of the energy encircled in the focal spot to that in the wings, can significantly change the interaction physics when the intensity in the wings fluctuates around the relativistic threshold, and this should be considered for accurate description of experimental results. Just like pulse energy, duration, peak intensity and temporal-intensity contrast, focal spot spatial-intensity contrast should be considered an important parameter to measure in an experiment and to communicate when reporting results. Our example measurements of proton acceleration demonstrate that it is another parameter in defining the optimisation and reproducibility of laser-driven radiation from solids, and thus impacts on the development of these sources towards wide-ranging applications.

## Methods

### Experiment

**Figure 6:**
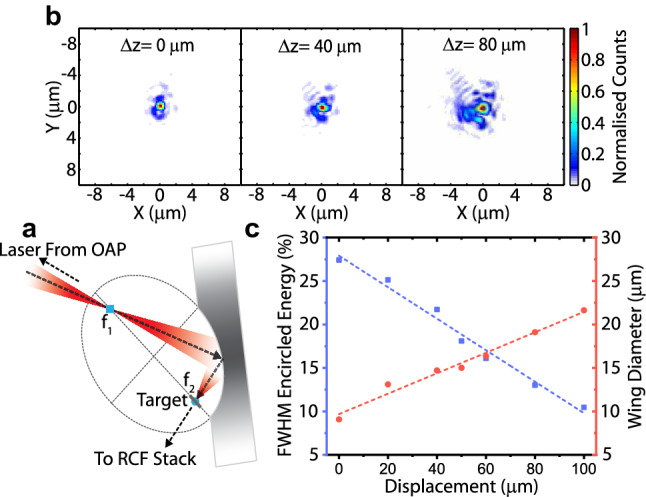
Focusing plasma mirror schematic and quantification of the focal spot wings. (**a**) Schematic of a focusing plasma mirror with the location of the target and direction radiochromic film (RCF) stack indicated. (**b**) Spatial-intensity distributions of the FPM output focal spot. (**c**) Encircled energy (blue) and spot wing diameter (red), as a function of OAP focus position displacement ($\Delta$z) from optimal (at $\hbox {f}_{{1}}$) along the laser propagation direction. Optimised alignment, i.e. $\Delta \hbox {z}=0\,\upmu \hbox {m}$, has values of $\phi =0.76 \,\upmu \hbox {m}$ and encircled energy= 27.4%.

The experimental measurements were performed using the Vulcan Petawatt laser at the Rutherford Appleton Laboratory. This laser provides *p*-polarised pulses of 1054 nm wavelength light, with energy up to 300 J on-target (after losses in the compressor and plasma mirror), and duration ($750\pm 100$) fs (FWHM). For the comparison between a *F*/1 focal spot and one of relativity larger size, two interchangeable plasma mirror (PM) configurations were deployed. The first uses an ellipsoidal focusing plasma mirror (FPM), depicted in Fig. 6a, which converts the standard *F*/3 off-axis parabola (OAP) focusing geometry used at the Vulcan Petawatt facility to *F*/1 focusing. Further details on the design and operation of this optic are provided in Refs.^13, 14^. The second uses a planar plasma mirror (PPM) to maintain the *F*/3 OAP focusing after reflection. Typically the PPM forms a $4.0\,\upmu \hbox {m}$ spot (FWHM), with an encircled energy of 28%, and the FPM, a $1.6 \,\upmu \hbox {m}$ spot (FWHM) at its output focus (position $\hbox {f}_{{2}}$), with a maximum measured encircled energy of 37%, when optimally aligned. Successful FPM operation (i.e. high quality focus formation) depends upon optimised alignment, reliant on the OAP focus spatially coinciding with the FPM input, position $\hbox {f}_{{1}}$. When used on a high power laser system, this condition is sensitive to variations in the OAP focus position (along its propagation direction), which occur on the tens of micron scale due to various effects, including thermal lensing in the laser chain altering the pulse divergence prior to focusing. This results in the focal spot position, nominally at $\hbox {f}_{{2}}$, being displaced, thus a target placed at $\hbox {f}_{{2}}$, will not be irradiated by the optimal focus, but one of lower encircled energy, due to an increase in the size and energy content of the spot wings, the degree of which is dependent on the magnitude of input focus displacement. The central spot size, however, remains relatively small in comparison to *F*/3 OAP focusing. The effect of non-optimised alignment on the FPM output focal spot is shown Fig. 6b, and quantified in Fig. 6c in terms of encircled energy and spot wing diameter (defined as the point the wings magnitude equals the background signal), as a function of input focus displacement from position $\hbox {f}_{{1}}$ ($\Delta$z). A lowering of spot FWHM encircled energy and an increase in wing size, is displayed as the misalignment magnitude increases, thus reducing the peak intensity of the FPM spot. These characterisation measurements were obtained using a testing set-up (described in Refs.^13, 14^), that utilises a 532 nm light source, and thus smaller spots/wings are obtained when compared to FPM usage on the Vulcan laser (operating at 1054 nm). As the effect of unoptimised FPM alignment results in decreased focal spot intensity and increased spot wing size (thus irradiation area), this gives us a method to measure not only the effect of spot size at intensities similar to those achieved by the larger spot ($\sim 10^{20}\, \hbox {W cm}^{-2}$, when used on the Vulcan laser), but crucially in this investigation the influence of focal spot spatial-intensity contrast, particularly in the case of tight focus, when the wings are relativistically intense. To quantify the magnitude of longitudinal displacements in the OAP focus position on full power laser shots, a Shack–Hartmann wavefront sensor was used to measure the degree of phase aberrations present in the laser wavefront, thus enabling the on-target intensity to be calculated^37^ and the focal spot wing conditions to be approximated. Measurements of proton acceleration, in both focusing geometry cases, were performed by irradiating $6\,\upmu \hbox {m}$ aluminium foils, with transverse dimensions of $2\times 2 \,\hbox {mm}$, at near-normal incidence. The spectral properties of the resultant beam of protons accelerated from the target rear were measured using a stack of $50\times 50 \,\hbox {mm}$ dosimetry film (radiochromic, RCF)^32^ positioned 50 mm behind the foil, centred on the target normal axis. Each layer of RCF is separated by an additional layer of known filter material and thickness. This enables the proton beam spatial dose distribution to be measured at discrete energy levels enabling the energy spectrum and beam divergence to be measured simultaneously.

### Simulations

Simulations were performed using the fully relativistic, 2D PIC code, EPOCH^36^. These consisted of a simulation box of size $75 \,\upmu \hbox {m}\times 230 \,\upmu \hbox {m}$ with $15,000 \times 23,040$ cells. The plasma was initialised as a $6 \,\upmu \hbox {m}$ thick slab of $\hbox {Al}^{11+}$ ions with a 20 nm-thick layer of protons at the target rear to approximate a contamination layer. This is neutralised by a population of electrons with a peak density of $660n_{crit}$ ($n_{crit}=m_e\epsilon _0\omega _L^{2}/e^2$, where $m_e$ is the electron rest mass and $\epsilon _0$ is the vacuum permittivity) corresponding to solid density aluminium and with an initial temperature of 10 keV. In order to approximate the degree of expansion induced by the amplified spontaneous emission (ASE), which arrives at the target in advance of the main laser pulse, a longitudinal exponential expansion was included at the front side with a scale length of $1\,\upmu \hbox {m}$, based on modelling using the HELIOS radiation-hydrodynamics code. We assume that the plasma expansion will be locally similar across the target in the region of the main focal spot and wings. It has been shown in experimental investigations of laser ASE that because it is generated differently to the main laser pulse, its distribution in the plane of the target is significantly larger than the central spot^38, 39^. Moreover, a simple calculation of the transverse expansion velocity of the resulting preplasma indicates that it covers a spatial region larger than the wings and central spot by the time that they arrive at the target.

The laser pulse was defined with a wavelength of 1054 nm, a Gaussian temporal profile with duration 400 fs (FWHM) and was focused to the front surface, as defined in the main text. The pulse duration is shorter than in the experiment (750 fs) due to restraints on the available computational resource. It was not possible to run the required number of simulations for the same duration as the pulses in the experiment. We have run test cases for a duration of 750 fs and find no significant difference when compared to the 400 fs-duration results. We also note that particle energies are exaggerated in 2D simulations (no energy dissipation out of the plane), resulting in an exaggerated degree of target expansion. A slightly shorter pulse in a 2D simulation thus more accurately models the expansion expected in the experiment. Finally, we note that test case simulations performed with 40 fs duration pulses (with and without wings) show changes in the overall energy coupling to fast electrons, but the conclusion on the role of the intensity in the wings remains unchanged. A more detailed investigation of the short pulse case will be the subject of a future study.

## Data Availability

Data associated with research published in this paper can be accessed at https://doi.org/10.15129/346d1e29-2f23-488f-b397-19237e1da1a4. Correspondence and requests for materials should be addressed to Paul McKenna (paul.mckenna@strath.ac.uk).
